# Establishment of Real-Time PCR Method to Differentiate *Phlebotomus sichuanensis* (Diptera, Psychodidae) from *P. chinensis* s.s. Based on Whole Mitochondrial Genome Analysis

**DOI:** 10.3390/life14121610

**Published:** 2024-12-05

**Authors:** Haowei Dong, Wenqi Shan, Hao Yuan, Qiuming Zhou, Wenbing Zhong, Maimaitijiang Wumaier, Kang Wang, Anjie Yang, Bing Rui, Hua Shi, Huiying Chen, Xiangyu Li, Yajun Ma, Heng Peng

**Affiliations:** 1 Department of Medical Pathogen Biology, College of Basic Medicine, Naval Medical University, Shanghai 200433, China; 2 College of Naval Medicine, Naval Medical University, Shanghai 200433, China; 3 Department of Vector Control, Haikou Center for Disease Control and Prevention, Haikou 570100, China; 4 Institute of Parasitic and Brucellosis Prevention and Treatment, Center for Disease Control and Prevention of Xinjiang Uygur Autonomous Region, Urumqi 830000, China; 5 Center for Disease Control and Prevention of Yangquan City, Yangquan 045000, China; 6 Chinese PLA Center for Disease Control and Prevention, Beijing 100071, China

**Keywords:** *Phlebotomus*, classification, mitochondrial genome, real-time PCR

## Abstract

*Phlebotomus sichuanensis*, considered a potential vector for visceral leishmaniasis (VL), is distributed in the southern Gansu and northern Sichuan regions in China. However, the high similarity in the morphology of *P. sichuanensis* and *P. chinensis* s.s. poses unresolved taxonomic challenges. In this study, phlebotomine sand flies were collected from three locations in the southern Gansu and northern Sichuan regions (SCB group) and three locations that are the dominant distribution areas of *P. chinensis* s.s. (ZHB group). Their whole mitochondrial genomes were sequenced and analyzed. The differential analysis revealed that there were 339 fixed differential sites in the mitochondrial genome-coding region of *P. chinensis* s.s. and *P. sichuanensis*, among which the *COI* gene had the most differential sites (57), followed by *ND5* (46), *ND4* (38), and *CYTB* (37), while *ATP8* had the least differential sites (4). The molecular genetic *p*-distance was calculated based on 13 protein-coding regions, and the genetic distance ranged from 0.001 to 0.018 in the ZHB group and from 0.001 to 0.006 in the SCB group, while the interspecies molecular genetic distance was 0.464–0.466 between the two groups. A phylogenetic maximum likelihood tree was constructed from 16 samples via tandem sequence of 13 protein-coding regions, and the topology showed that the ZHB and SCB groups formed separate clusters. A real-time PCR method was established based on the differences in the COI fragment, which can identify *P. sichuanensis* from *P. chinensis* s.s. effectively. This study presents objective evidence of the genetic differentiation between *P. sichuanensis* and *P. chinensis* s.s., and provides a method for identifying these two morphologically highly similar VL-transmitting sandflies.

## 1. Background

Sand flies (Diptera, Psychodidae, Phlebotominae) are hematophagous insects that pose a threat to human health by transmitting diseases such as leishmaniasis, phlebotomus fever, and Bartonella disease [1]. Visceral leishmaniasis (VL) was once a prevalent parasitic disease in China [2]. However, the implementation of disease control measures has significantly reduced the number of cases [3]. Since the beginning of the 21st century, the incidence of visceral leishmaniasis in China has remained low [4]. Nevertheless, recent years have witnessed an increase in visceral leishmaniasis cases in China due to changes in natural ecology and social environment dynamics [5].

Four sand fly species have been identified as vectors for visceral leishmaniasis in China: *Phlebotomus chinensis* s.s., *P. longiductus*, *P. wui*, and *P. alexandri* [6]. Among these species, *P. chinensis* s.s. is primarily widely distributed across 15 provinces (municipalities/autonomous regions) north of the Yangtze River, as well as Yunnan, Guizhou, Guangdong, and southern Jiangsu province. It is considered the most significant vector for visceral leishmaniasis in China [7]. However, morphological variations have been observed among *P. chinensis* s.s. populations within certain regions, and their taxonomy remains an unresolved issue.

The incidence of visceral leishmaniasis is high in southern Gansu and northern Sichuan, which are regions characterized by a mountainous canyon landform with significant altitude variation. Prior to the 1980s, *P. chinensis* s.s. was considered the dominant species in this region. However, Leng et al. (1983) reported the discovery of *P. sichuanensis* as a new species in this area for the first time [8]. Through a comparative analysis of the morphology, life cycle, and isozyme spectra between *P. sichuanensis* and *P. chinensis* s.s., they concluded that *P. sichuanensis* predominates in areas above an altitude of 900 m, while *P. chinensis* s.s. is distributed in areas below 900 m in altitude, although there may be some limited overlap in their vertical distribution ranges [9,10]. Furthermore, Leng et al. suggested that, apart from southern Gansu and northern Sichuan, *P. sichuanensis* could also be found in high-altitude areas across Southwest China, including Sichuan, Gansu, Tibet, Yunnan, and Qinghai provinces [11,12].

However, Xiong et al. presented different views on the taxonomic status of *P. sichuanensis*. They argued that based on morphological observations and life history studies, the “*P. sichuanensis*” species described by Leng et al. [8,9,10,11,12] is synonymous with *P. chinensis* s.s., while Xiong et al. also noted that male sand fly specimens from high-altitude regions exhibit body size variations and antennal patterns that overlap with those observed in *P. chinensis* s.s. from plain areas. Additionally, body size measurements, life cycle assessments after feeding in the laboratory, and cross-breeding experiments all indicated similarities between *P. sichuanensis* and *P. chinensis* s.s. [13]. Therefore, Xiong et al. proposed that *P. sichuanensis* should be regarded as a synonymous rather than a distinct species within the genus of *Phlebotomus* [14].

Although taxonomy based on morphology is important for the classification of sandflies, it has been shown to be insufficient for the delimitation of some taxa [15,16]. Due to the challenges in determining the taxonomic status of *P. sichuanensis* based on morphology alone, molecular methods have been employed to study its molecular characteristics. Zhao et al. utilized scanning electron microscopy and random amplified polymorphic DNA polymerase chain reaction (RAPD-PCR) to analyze five groups of sand flies collected from Shanxi, Shaanxi, and Sichuan, and they concluded that *P. sichuanensis* and *P. chinensis* s.s. should be considered the same species [17]. In contrast, through an analysis of the microsatellite loci and *CYTB* sequence of *P. sichuanensis*, Zhang et al. found differences in the molecular characteristics between *P. chinensis* s.s. and *P. sichuanensis* to some extent [18,19]. Moreover, genetic differentiation was observed within the population of *P. chinensis* s.s. itself, suggesting a close relationship between these two species but also supporting the classification of *P. sichuanesis* as an independent species [20]. However, these studies provided limited evidence due to technological limitations.

Significant advancements in gene sequencing technology in recent years, along with its reduced costs, allowed us to compare the whole mitochondrial genome sequences of *P. chinensis* s.s. and *P. sichuanensis*. In this study, samples of sand flies were collected from areas above an altitude of 900 m in southern Gansu and northern Sichuan, together with samples collected from Beijing, Shanxi, and Shaanxi. Morphological observation and analysis of whole mitochondrial genome sequences were conducted to confirm the taxonomic status of “*P. sichuanensis*”.

## 2. Methods

### 2.1. Sample Collection

**Figure 1:**
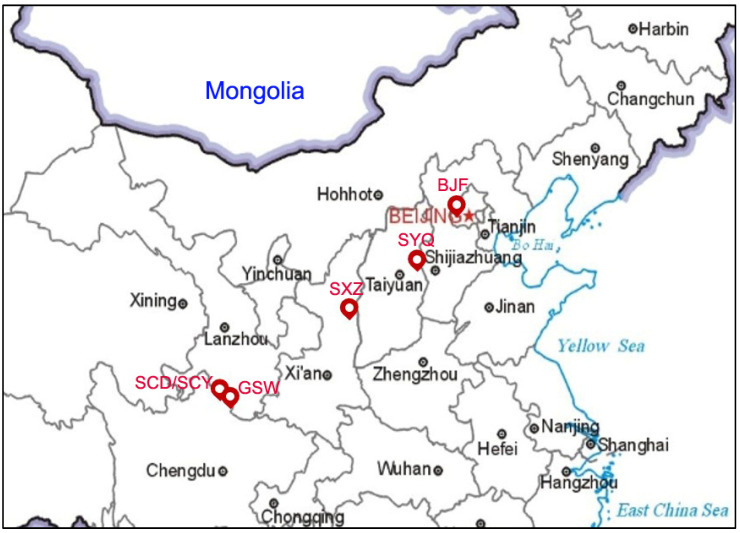
Schematic map showing the collection sites. Note: BJF, Fangshan District, Beijing City; SYQ, Yangquan City, Shanxi Province; SXZ, Yichuan County, Shaanxi Province; GSW, Wen County, Gansu Province; SCD/SCY, Jiuzhaigou County, Sichuan Province. The red star indicate the capital of China.

The sand fly samples used in this study were collected from six sites across five provinces or cities of China between 2009 and 2023 in different habitats such as caves, cattle stalls, chicken houses, and donkey stalls (Figure 1, Table 1). Sand fly adults were caught using insect light traps (Houji Dianzi, Shen Zhen, China) and an artificial aspirator from dusk to dawn. The collection of sand flies from livestock corrals was conducted with the consent of the owners. After being anesthetized with chloroform, the sand fly adult samples were dried and stored in a 1.5 mL centrifuge tube individually, brought back to the laboratory, and stored in a −20 °C cryogenic freezer.

### 2.2. Morphology Observation

The sand fly samples were rinsed with 75% alcohol, their external morphological feature was observed, and the body length was measured under a stereo microscope (Olympus, SZX2FOF, Tokyo, Japan). Then, the head and the posterior segment of the abdomen of these sand flies were cut off and transferred into 0.8 mL centrifuge tubes containing 10% KOH solution to digest overnight at 37 °C. The remaining parts of each sample were stored individually in 1.5mL centrifuge tubes for genomic DNA extraction. After digestion, the head and posterior segment of the abdomen were mounted on a glass slide. The morphological characteristics of the cibarium, pharynx, spermathecae, and male genitalia were dissected under a stereo microscope and observed under a binocular microscope (Leica, DM500, Wetzlar, Germany), according to the methods used by Xiong [21]. Samples with the morphological characteristics of *P. chinensis* s.l. were selected for subsequent studies. The antennal patterns of male sand flies were observed to investigate if there were morphological differences between *P. sichuanensis* and *P. chinensis* s.s. as previously reported. Moreover, the wing length was measured and the wing/body length ratio was calculated.

### 2.3. DNA Extraction and Sequencing

The genomic DNA of the sand flies was extracted using the Qiagen Micro DNA kit (Qiagen, Hilden, Germany), following the manufacturer’s instructions. The extracted genomic DNA concentrations were quantified using the Qubit method. The genomes of the sand fly samples were used for library construction and next-generation sequencing. The genomes were fractionated into 300–500 bp fragments using Covaris M220 (Covaris, Woburn, MA, USA) to construct the libraries. The constructed libraries were then sequenced using the Illumina Novaseq 6000 platform (Illumina, San Diego, CA, USA) with S4 channel and 150bp paired-end sequencing.

### 2.4. Genomic Assembly and Annotation

The original image data obtained via Illumina sequencing were converted into FASTQ-format sequence data via base calling in order to obtain the original sequencing data file. Using Fastp v.0.23.3 with the default parameters, the raw sequencing data of each sample were evaluated for quality using various statistics, including the base content and base quality distribution statistics, and the obtained data were further checked for their quality and validated [22]. The de novo method was used to splice the read sequences obtained through multiple iterations using the NOVOPlasty online software (https://github.com/ndierckx/NOVOPlasty, accessed on 6 May 2023) [23]. Based on the de novo assembled mitochondrial genome sequence, gene annotation was performed using the annotation module of MitoZ (https://github.com/linzhi2013/MitoZ, accessed on 12 July 2023) and visualized using the visualization module [24]. Finally, all mitochondrial genome sequences were manual verified using Geneious v.11.0 software.

### 2.5. Analysis of Mitogenome Characteristics and Variation Sites

The coding regions and the rRNA and tRNA sequences were extracted using Phylosuite software v1.2.3 for sequence alignment, trimming, and splicing [25]. The nucleotide composition of each mitochondrial genome was counted using Phylosuite. The AT skew and GC skew of each gene fragment were calculated using the following formulas: AT skew = (A% − T%)/(A% + T%) and GC skew = (G% − C%)/(G% + C%). The secondary structure of tRNAs was predicted using the online tool MITOS webserver (http://mitos.bioinf.uni-leipzig.de/, accessed on 1 August 2023) [26]. The relative uniform codon usage (RSCU) of each mitochondrial genome-coding region was calculated using MEGA XI v.0.13 software [27]. Pairwise *p*-distances of species were calculated based on 13 protein-coding genes (PCGs) using MEGA XI software. The coding region sequences were aligned with MEGA XI software to screen out the differential sites. The differential locus analysis was performed based on 13 protein-coding regions of the mitochondrial genome, and only parsimony-informative sites were counted as differential loci.

### 2.6. Phylogenetic Analysis

The mitochondrial genome sequences used in the phylogenetic analysis were derived from 16 samples collected for this study and 3 sand fly mitochondrial genome sequences obtained from the GenBank database (https://www.ncbi.nlm.nih.gov/GenBank/, accessed on 11 August 2023), namely, *P. chinensis* (KR349297), *P. papatasi* (NC028042), and *Lutzomyia trinidadensis* (KX356037). The best partitioning scheme and evolutionary models were selected using PartitionFinder2 v2.1.1, with the greedy algorithm under the corrected Akaike information (AICc) criterion [28]. Afterward, a phylogenetic tree was constructed using IQ-TREE v.2.2.0 software based on the maximum likelihood method with bootstrapping values defined from 5000 repetitions [29]. The topological structures were viewed and visualized with MEGA XI software, and the graphs were edited using the online tool ITOL (https://itol.embl.de/itol.cgi, accessed on 24 October 2023) [30].

### 2.7. Real-Time PCR Method to Distinguish P. chinensis s.s. and P. sichuanensis

Based on the results of the differential site analysis, the *COI* sequence was selected as the target sequence for the molecular identification of *P. chinensis* s.s. and *P. sichuanensis* species. The Primer-BLAST online tool (https://www.ncbi.nlm.nih.gov/tools/primer-blast/, accessed on 13 October 2023) was used to find species-specific primers and manually adjust them. The primers used to identify *P. chinensis* s.s. were the forward primer *COI*-ZHF1 (5′-GGGCTAAACTTAAACTCCTATC-3′) and the reverse primer *COI*-ZHR1 (5′-GTTGGGGGAAAAAGGTTAGAT-3′). The primers used to identify *P. sichuanensis* were the forward primer *COI*-SCF4 (5′-GTATATCCCCCTCTTTCTAG-3′) and the reverse primer *COI*-SCR4 (5′-CCTAAAATGGATGAAACCCC-3′). These primers were synthesized by Biosune Biotechnology (Shanghai, China). The genomic DNA of *P. chinensis* s.s. and *P. sichuanensis* were used to detect the sensitivity and optimize real-time PCR reaction conditions. In addition, the genomic DNA of *P. longiductus*, *P. wui*, and *P. alexandri* preserved in our laboratory were used for specific evaluation. The real-time PCR was conducted on a LightCycler^®^ 480 Instrument II (Roche, Basel, Switzerland) using ChamQ SYBR qPCR Master Mix (Vazyme, Nanjing, China) following the manufacturer’s instructions. The optimized reaction conditions involved a pre-heating step at 95 °C for 10 s, and the PCR consisted of 40 cycles of denaturation at 95 °C for 10 s, annealing at 62 °C for the *P. sichuanensis*-specific primer or at 59 °C for the *P. chinensis* s.s.-specific primer for 30 s, and extension at 72 °C for 10 s, with a final cooling step at 40 °C for 30 s. Fluorescent signals were detected during the extension phase of each cycle. The fluorescence threshold and Ct value were calculated automatically using LightCycler^®^ 480 software, and Ct ≤ 26.00 was set as the cut-off value.

## 3. Results

### 3.1. Morphological Identification

In this study, a total of 137 sand flies were sampled from six collection sites. The collection sites included Wen County in Gansu Province (GSW) and Jiuzhaigou County in Sichuan Province (SCD and SCY) in the northern Sichuan region, with altitudes ranging from 1296 to 2153 m; these areas were considered the typical distribution areas of *P. sichuanensis* by Leng et al. [6]. These samples were grouped into SCB in this study. The other three collection sites were Fangshan District of Beijing (BJF), Yangquan City of Shanxi Province (SYQ), and Yichuan County of Shaanxi Province (SXZ), with altitudes ranging from 180 to 1021 m; these areas are the dominant distribution areas of *P. chinensis* s.s., and the samples were grouped into ZHB in this study. Among the distribution areas, BJF is the type locality of *P. chinensis* s.s. All samples were identified as *P. chinensis* s.l. (including *P. sichuanensis*) based on their external and internal morphological characteristics.

There were no significant differences in female spermathecae between the SCB and ZHB specimens. Among the 29 male sand flies in the SCB group, two samples had the antennal patterns of 2/III-VIII and 1/IX-XV, while the rest had the antennal pattern of 2/III-XV. The antennal patterns of the 16 ZHB sand flies were all 2/III-XV.

The male body size and wing length of *P. chinensis* s.s. and *P. sichuanensis* are controversial points, so the male samples were measured. We measured 29 male sand flies in the SCB group. The average body length was 3034.1 μm, the average wing length was 2095.0 μm, and the body length/wing length ratio was 1.45 (1.28–1.69). Sixteen male sand flies from the ZHB group were measured. The average body length was 3361.5 μm, the average wing length was 2192.5 μm, and the average body length/wing length ratio was 1.54 (1.27–1.75). There was no significant difference in wing length between the two groups (*p* = 0.146). The difference in the ratio of body length to wing length was statistically significant (*p* = 0.007), but the value intervals mostly overlapped.

### 3.2. Composition and Structure of the Mitochondrial Genome

#### 3.2.1. Overview of Mitochondrial Genome Structure and Base Composition

The whole mitochondrial genome sequences (GenBank numbers OR806948-OR806963) of 16 sand flies were obtained, including seven samples from the SCB group and nine samples from the ZHB group. The mitochondrial genome length of the SCB group was 15,161–16,284 bp, with an average length of 15,441 bp. The mitochondrial genome length of the ZHB group was 15,288–15,816 bp, with an average length of 15,410 bp. The difference in length is mainly due to the A+T-rich control region, which is difficult to determine accurately due to the peculiarity of the base composition.

**Figure 2:**
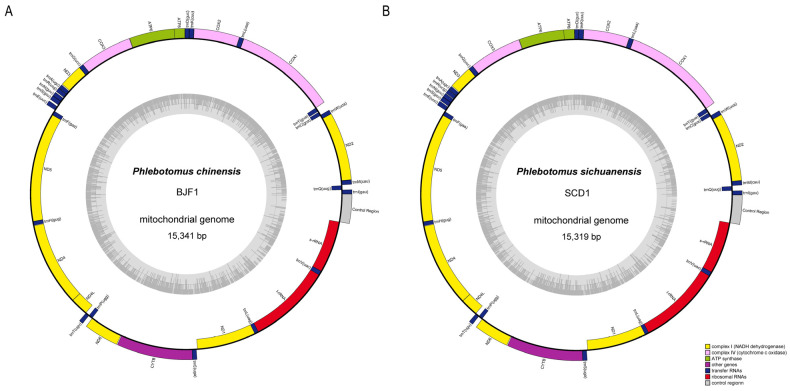
Structures of the mitochondrial genomes of *Phlebotomus sichuanensis* (**A**) and *P. chinensis* (**B**). The gray inner ring represents the GC content pattern.

The mitochondrial genome contains 37 genes, including 13 protein-coding genes, 2 rRNA genes, and 22 tRNA genes, in addition to a control region. Of these, 23 genes are located in the heavy chain and 14 genes are located in the light chain (Figure 2). These genes are consistent with the number and sequence of the reported mitochondrial genomes of *P. chinensis* (GenBank number: KR349297) and *P. papatasi* (GenBank number: NC028042).

The mean values of ATCG base content in the mitochondrial genome of the seven sand flies from the SCB group were 39.0%, 39.8%, 13.0%, and 8.2%, and the mean values of AT and GC skews were −0.010 and −0.224, respectively. The mean values of ATCG base content in the mitochondrial genome of the nine sand flies from the ZHB group were 38.9%, 39.4%, 13.3%, and 8.3%, and the mean values of AT and GC skews were −0.007 and −0.230, respectively (Supplementary File S1). The mitochondrial genomes showed a strong preference for A and T, with the highest content of A+T in the control region (94.7% in the SCB group and 92.6% in the ZHB group). The second codon position of PCGs had the lowest A+T content, with 67.9% in the SCB group and 67.8% in the ZHB group, which was similar to the mitochondrial genome characteristics of other Diptera insects.

#### 3.2.2. Genetic Characteristics of Coding Regions

##### Gene Length of Coding Regions

Among the 13 coding region genes, the *ND1*, *ND4*, *ND4*L, and *ND5* genes were located in the light chain, while the other genes were located in the heavy chain (see Supplementary File S2). The total length of the protein-coding region of the mitochondrial genome for both groups was 11,194 bp. Except for *ND2* and *ND6*, the length of each of the other protein-coding regions was the same, including *ATP6* (678 bp), *ATP8* (162 bp), *COI* (1534 bp), *COII* (684 bp), *COIII* (789 bp), *CYTB* (1140 bp), *ND1* (939 bp), *ND3* (354 bp), *ND4* (1333 bp), *ND4*L (288 bp), and *ND5* (1740 bp). The *ND2* gene in the ZHB group (1026 bp) had one more codon AAT before the stop codon compared with the *ND2* gene in the SCB group (1023 bp). The *ND6* gene in the SCB group (528 bp) had one more codon A(C)AT before the last base A compared with the *ND6* gene in the ZHB group (525 bp).

##### Base Composition of Coding Regions

The AT-skew value of each coding gene in both groups was negative. The *ND4L* gene had the lowest value of −0.284 in the ZHB group and −0.278 in the SCB group. The *COII* gene had the highest AT-skew value of −0.086 in the ZHB group and −0.081 in the SCB group. The GC-skew values were positive for *ND1*, *ND4*, *ND4L*, and *ND5*, and the other nine genes had negative GC-skew values. In the ZHB group, the highest GC-skew value was observed for the *ND4*L gene (0.434) and the lowest was observed for the *ATP8* gene (−0.580). In the SCB group, the *ND4* gene had the highest value (0.374) and the *ATP8* gene had the lowest value (−0.586) (see Supplementary File S1).

##### Start and Stop Codons

The start and stop codons of the 13 protein-coding genes in the ZHB and SCB groups are almost consistent. Among them, the *ATP6*, *COII*, *COIII*, *CYTB*, *ND4*, and *ND4*L genes use ATG as the start codon, and the *ND2*, *ND5*, and *ND6* genes use ATA as the start codon. Moreover, the *ND1* and *ND3* genes use ATT and ATC as the start codon, respectively. It is worth noting that the *ATP8* gene uses ATC as the start codon in the ZHB group, while ATT is the start codon in the SCB group. In addition, the TCG codon is designated as the *COI* start codon because the hexanucleotide ATTTAA next to it is involved in start signaling; this finding has also been reported in other Diptera insects. The ZHB and SCB groups are consistent in terms of stop codons for the coding genes. Most genes use TAA as the stop codon, while only the *CYTB* gene uses the TAG codon. The *COI* and *ND4* genes use a single base T as the stop codon (see Supplementary File S2). Incomplete stop codons are commonly found in insect mitochondrial genomes and are presumed to be converted to ‘TAA’ through post-transcriptional modifications such as polyadenylation.

##### Amino Acid Composition Characteristics

Except for the stop codon, 3719 amino acids are encoded in the 13 protein-coding regions. Leu is the most abundant amino acid, followed by Ile, Phe, and Ser, while Cys is the least abundant. The codon usage of the protein-coding genes was estimated based on the RSCU values. The codons with the highest frequency among the 13 PCGS are TTA of Leu, AUU of Ser, CGA of Arg, and CCT of Pro (see Supplementary File S3). Similar to the coding regions of other insect mitochondrial genomes, the third codon of the protein-coding region prefers to use A/T, and the RSCU value of most codons ending in A/T is greater than 1, while the RSCU value of the third codon G/C is less than 1.

#### 3.2.3. Non-Coding Gene Structure

The average sequence length of 12S rRNA and 16S rRNA was 823 bp and 1372 bp in the ZHB group, and 828 bp and 1369 bp in the SCB group, respectively. The two RNA genes are located between the *tRNA Leu* (TAG) gene and the control region, and they are separated by the *tRNA Val* gene. The A+T content of the rRNA gene in the two groups was about 84.0%. In addition, the A+T- and G+C-skew values of the tandem nucleotide sequences of the two rRNA genes were almost 0. The tRNA genes were distributed throughout the mitochondrial genome and ranged in length from 62 to 71 bp. Among the secondary structures predicted by 22 tRNA genes, only *tRNA Ser* (GCT) lacked the dihydrouridine (DHU) arms, and the remaining tRNA genes all formed typical clover structures (Supplementary File S4). This atypical tRNA secondary structure has been reported in many insects. The tandem nucleotide sequences of the 22 tRNAs showed a high A+T preference, accounting for 80.5%, as well as a negative AT bias and a positive GC bias (Supplementary File S1).

### 3.3. Differential Site Analysis

There were 339 location differences in the 13 protein-coding regions of the mitochondrial genome between the ZHB and SCB groups, among which the *COI* gene had the highest number of differential sites (57), followed by *ND5* (46), *ND4* (38), and *CYTB* (37), while *ATP8* had the least differential sites (4). The highest base variation rate was found for *ATP6* (4.1%), followed by *ND3* (4.0%), *COIII* (3.9%), and *COI* (3.7%), and the lowest was for *COII* (1.8%) (Table 2). These differential sites are mainly distributed in the third base of the codon, and most of them cause synonymous mutations. The differences in the amino acids encoded by the mitochondrial genomes of the ZHB and SCB groups were small, with only 35 differences, among which the *ND5* gene had the largest difference of nine amino acids, followed by *ND6* (5), *CYTB* (4), *COIII* (3), *ND2* (3), and *ND4* (3). There was no difference in the amino acids encoded by the *ATP8* and *ND4*L genes (Supplementary File S5).

### 3.4. Phylogenetic Analysis

The molecular genetic *p*-distance was calculated based on the 13 protein-coding regions, and the results showed that the genetic distance ranged from 0.001 to 0.018 in the ZHB group and from 0.001 to 0.006 in the SCB group. The molecular genetic distance between the ZHB group and SCB group was 0.464–0.466. The interspecific differences were obviously larger than the intraspecific differences. The genetic distance among the geographic populations of the ZHB group was 0.001–0.018 for BJF, 0.001–0.012 for SYQ, and 0.007–0.012 for SXZ. The genetic distance among the geographic populations of the SCB group was 0.006 for GSW, 0.002–0.004 for SCD, and 0.002 for SCY (Supplementary File S6).

**Figure 3:**
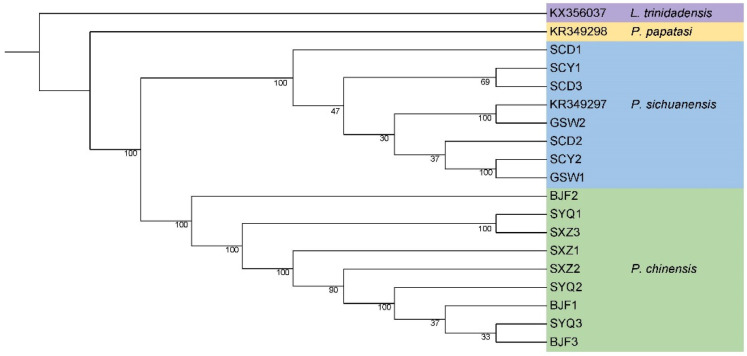
Phylogenetic reconstruction via maximum likelihood based on the 13 PCG tandem sequences, with bootstrap analysis of 1000 replications. The values of bootstrapping support are shown to the left of the branch point. The tandem sequences of the 13 PCGs of *L. trinidadensis* and *P. papatasi* were extracted from GenBank as an outgroup.

A phylogenetic maximum likelihood tree was constructed from 16 samples via tandem sequencing of the 13 protein-coding regions. The topology showed that the ZHB group and the SCB group formed separate clusters. Samples from the same collection site were not clustered together; rather, the samples were clustered across sites, showing small geographic population differences. The mitochondrial genome sequences of *P. chinensis* published in GenBank were clustered together with the nine sand flies in the ZHB group in this study, but they were distinguished from the sand flies in the SCB group (Figure 3).

Based on differential site analysis and phylogenetic analysis of mitochondrial genomes, there are significant genetic differences between the SCB and ZHB groups, providing evidence that they are two different species. The sand flies in the ZHB group are *P. chinensis* s.s., while the sand flies in the SCB group are *P. sichuanensis*.

### 3.5. Molecular Identification

The real-time PCR method was used to verify the sand fly samples collected in the field. The results showed that the primers *COI*-ZHF1 and *COI*-ZHR1 could specifically detect 38 *P. chinensis* s.s. samples from three collection sites, with an average Ct value of 20.42, while the detection of 38 *P. sichuanensis*, 5 *P. longiductus*, 5 *P. wui*, and 5 *P. alexandri* samples was negative. The primers *COI*-SCF4 and *COI*-SCR4 could specifically detect 38 *P. sichuanensis* samples from three collection sites, with an average Ct value of 18.63, while the detection of 38 *P. chinensis* s.s., 5 *P. longiductus*, 5 *P. wui*, and 5 *P. alexandri* samples was negative (Supplementary File S7). Overall, this method can be used to differentiate *P. chinensis* s.s. and *P. sichuanensis* from the common sand fly species transmitting visceral leishmaniasis in China.

## 4. Discussion

The debate over the taxonomic status of *P. sichuanensis* has been ongoing for four decades. Due to limitations in techniques, conclusive evidence could not be provided in previous studies. In this study, sand fly samples were collected from six different sites and analyzed for morphological and whole mitochondrial genome characteristics.

According to Leng et al., the antennal pattern of male *P. sichuanensis* sand flies is 2/III-VIII, 1/IX-XV, which is an important morphological feature. In this study, we examined 29 male sand flies collected from areas above 900 m in altitude in southern Gansu and northern Sichuan, of which only two matched the description. This suggests that the antennal pattern is not a stable hereditary characteristic for *P. sichuanensis*. Additionally, the body length and body length/wing length ratio of *P. sichuanensis* and *P. chinensis* s.s. showed considerable overlap, making it difficult to use these as reliable morphological characteristics for species identification. Similarly, no obvious differences were found between the female spermathecae segments, male aedeagus tubercle position, or antennal segment lengths between these two kinds of sand flies. In summary, it is challenging to identify *P. sichuanensis* from *P. chinensis* s.s. based solely on morphology.

The complexity of sand fly taxonomy and systematics underlines the necessity of integrative tools. Several molecular markers have been employed to assess the molecular taxonomy of sand flies, including the mitochondrial DNA such as *COI*, *CYTB*, *ND1*, *ND4*, *ND5* genes, in addition to both the small and large ribosomal RNA subunits (12S rRNA and 16S rRNA) [31].

To date, studies on the molecular characterization of sand flies in China are relatively scarce. Chen et al. suggested that the mitochondrial genome *COI* and *CYTB* sequences can serve as ideal molecular markers for sand fly identification [32]. Mitochondria strictly inherit their mtDNA genomes maternally, which contain abundant genetic and phylogenetic population research information with high point mutation rates in gene sequences. Mitochondrial genome sequencing has been widely employed for insect identification [33,34]. Therefore, we determined the mitochondrial genomes of the sand flies collected from various regions across China, including from both the model habitat and absolute dominant area of *P. chinensis* s.s., along with the dominant area of *P. sichuanensis* identified by Leng et al. The differential site analysis revealed fixed differences in 13 protein-coding regions, where the significantly different gene fragments included the *COI*, *ND5*, *ND4*, and *CYTB* genes. The phylogenetic analysis demonstrated that the interspecific genetic distance between *P. sichuanensis* and *P. chinensis* s.s. was considerably greater than the intraspecific distance; furthermore, these two species clustered separately within the evolutionary tree structure. Thus, in this study, we provide promising molecular evidence supporting *P. sichuanensis* as an independent species from *P. chinensis* s.s.

It is worth noting that, in the construction of the phylogenetic tree, we initially incorporated the mitochondrial genome sequence (KR349297) of “*P. chinensis*” in GenBank reported by Ye et al. in 2015 [35]. Our analysis revealed that this sample clustered with *P. sichuanensis* and was distinct from *P. chinensis* s.s. This sand fly specimen was collected from Wen County, Gansu Province, which is situated within the southern Gansu and northern Sichuan regions. The comparative analysis showed a high similarity between the KR349297 and *P. sichuanensis* mitochondrial genome sequences. Considering these results, it is more appropriate to rename the sequence labeled as KR349297 to correspond to its true identity as belonging to *P. sichuanensis*.

Due to the challenges associated with morphological identification, we developed a RT-PCR method for differentiating *P. sichuanensis* from *P. chinensis* s.s. Based on the *COI* sequence analysis, we successfully designed species-specific primers for both *P. chinensis* s.s. and *P. sichuanensis*, enabling their differentiation from other sand fly species that are responsible for transmitting visceral leishmaniasis in China using real-time PCR. The establishment of this molecular identification method enabled the investigation of the role of *P. sichuanensis* in disease transmission.

Because of the limited number of samples, all *P. sichuanensis* samples used in this study were collected from altitudes above 900 m, with two female samples collected at higher altitudes (>2000 m), resulting in a lack of morphological data for male samples from low altitudes. Further exploration is needed to understand the distribution of sand flies below 900 m in the southern Gansu and northern Sichuan regions, which were previously believed to be overlapping regions for both *P. chinensis* s.s. and *P. sichuanensis* distributions.

## 5. Conclusions

In this study, we provided promising molecular evidence supporting species-level differences in the mitochondrial gene sequences between *P. sichuanensis* and *P. chinensis* s.s. Furthermore, a reliable real-time PCR method was established for distinguishing *P. sichuanensis* from *P. chinensis* s.s., which provides a potent tool for research on the role of *P. sichuanensis* in disease transmission.

## Figures and Tables

**Table 1:** Information on the sand fly samples collected in the present study.

Code	Collection Sites	Date	Altitude (m)	Coordinate	No. of Sample
BJF	Fangshan District, Beijing City, China	2023.06	180	115°59′ E, 39°66′ N	8♀
SYQ	Yangquan City, Shanxi Province, China	2017.06	837	113°56′ E, 38°01′ N	24♀, 10♂
SXZ	Yichuan County, Shaanxi Province, China	2009.07	1021	110°14′ E, 36°31′ N	15♀, 6♂
GSW	Wen County, Gansu Province, China	2009.07	1296	104°25′ E, 33°18′ N	19♀, 15♂
SCD	Jiuzhaigou County, Sichuan Province, China	2009.07	2153	104°15′ E, 33°14′ N	5♀
SCY	Jiuzhaigou County, Sichuan Province, China	2009.07	1503	104°15′ E, 33°14′ N	21♀, 14♂

**Table 2:** Statistics of the differential sites in the 13 coding regions between *P. chinensis* s.s. and *P. sichuanensis*.

Gene	Length (bp)	Number of Differential Sites	Proportion of Differential Sites
*ATP6*	678	28	4.13%
*ATP8*	162	5	3.09%
*COX1*	1534	57	3.72%
*COX2*	684	12	1.75%
*COX3*	789	31	3.93%
*CYTB*	1140	37	3.25%
*ND1*	945	20	2.12%
*ND2*	1026	27	2.63%
*ND3*	354	14	3.95%
*ND4*	1335	38	2.85%
*ND4L*	288	8	2.78%
*ND5*	1740	46	2.64%
*ND6*	525	16	3.05%
All PCGs	11,200	339	3.03%

## Data Availability

The genomic data provided and used in the article can be obtained from GenBank. For further inquiries, please directly contact the corresponding author.

## Supplementary Materials

The following supporting information can be downloaded at: https://www.mdpi.com/article/10.3390/life14121610/s1, File S1: Information on the AT/GC contents and AT/GC skews of the obtained mitochondrial genomes; File S2: Gene position, gene length, and the start and end codons of the obtained mitochondrial genomes; File S3: Relative synonymous codon usage (RSCU) of *P. chinensis* s.s. and *P. sichuanensis* mitochondrial DNA. The RSCU values are presented on the y-axis, and the families of synonymous codons and their respective amino acids are presented on the x-axis. Colored squares indicate the difference in the third base of the codon; File S4: Secondary structures of the tRNAs obtained in this study; File S5: Detail of the differential sites in the 13 PCGs between *P. chinensis* s.s and *P. sichuanensis* obtained in this study; File S6: The pairwise distance between *P. chinensis* s.s. and *P. sichuanensis* based on 13 PCGs; File S7: Ct value of each sample in species-specific primer validation.
