# Supplementary figures and images for: Establishment of Real-Time PCR Method to Differentiate Phlebotomus sichuanensis (Diptera, Psychodidae) from P. chinensis s.s. Based on Whole Mitochondrial Genome Analysis
Source: Life (Basel). 2024 Dec 5;14(12):1610. doi: 10.3390/life14121610 (PMC11676697; doi:10.3390/life14121610)

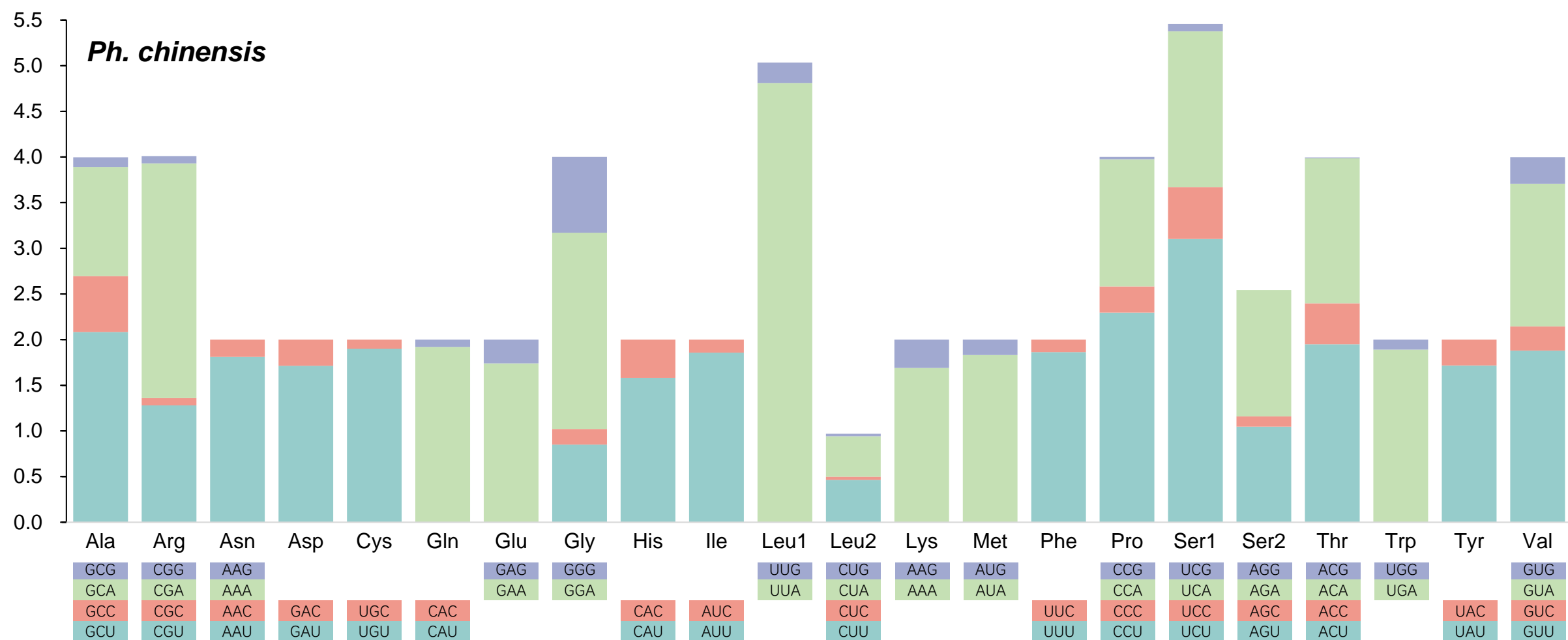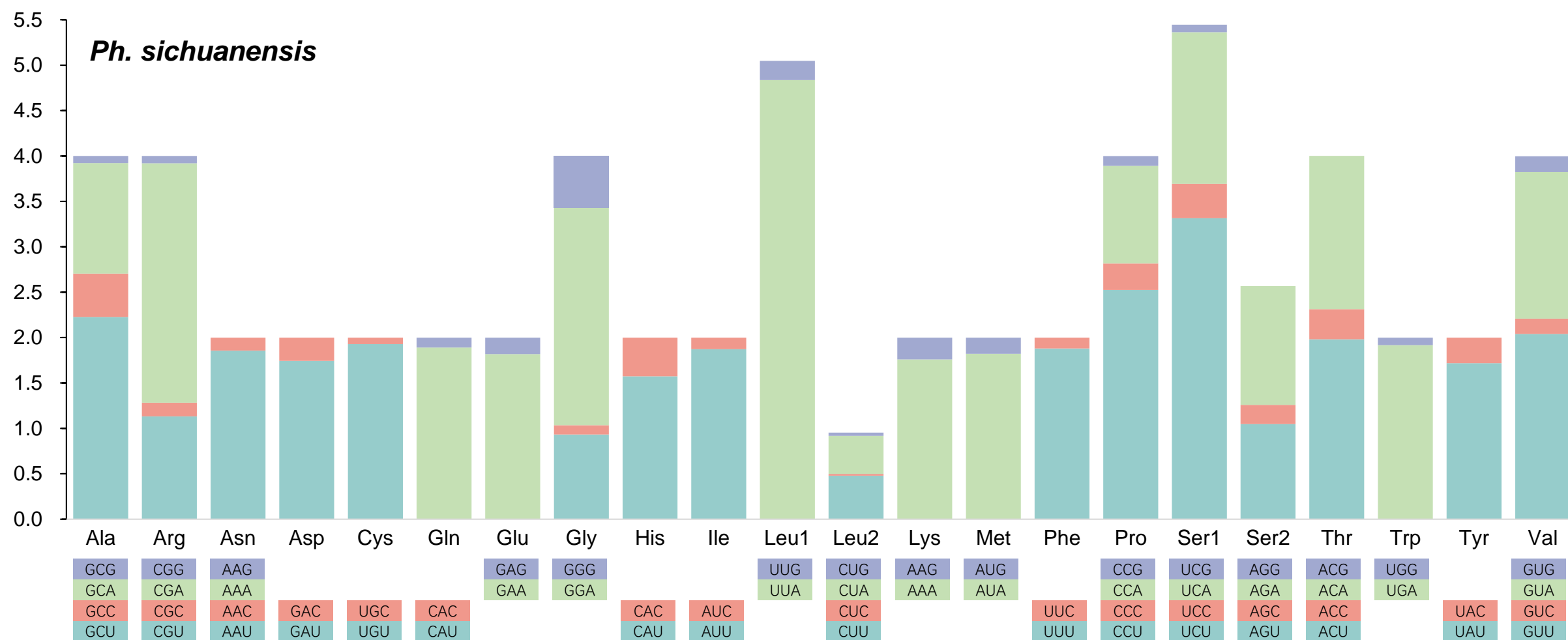

Supplement: Supplementary file 1 [file life-14-01610-s001.zip › Supplementary File 3.pdf]

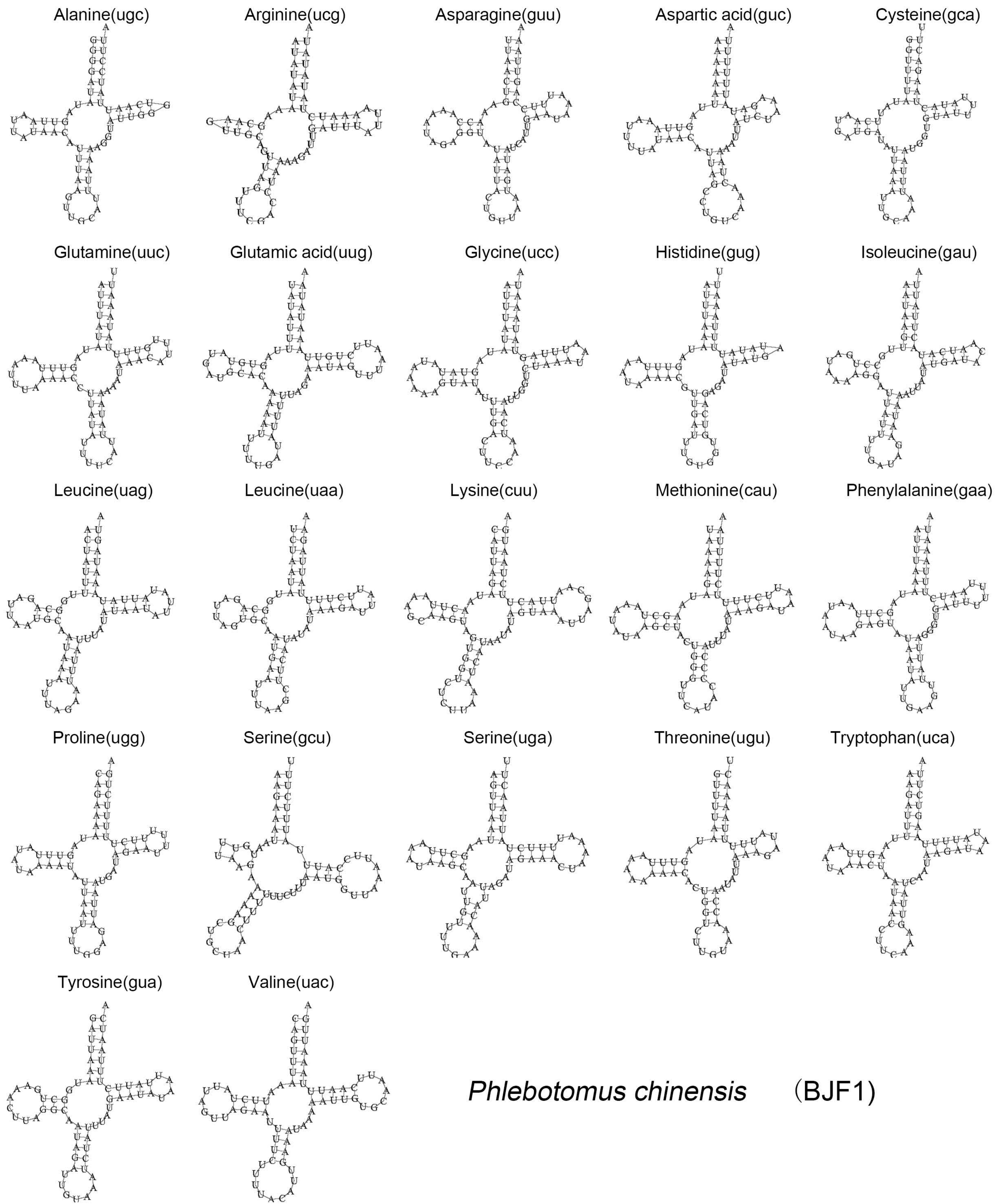

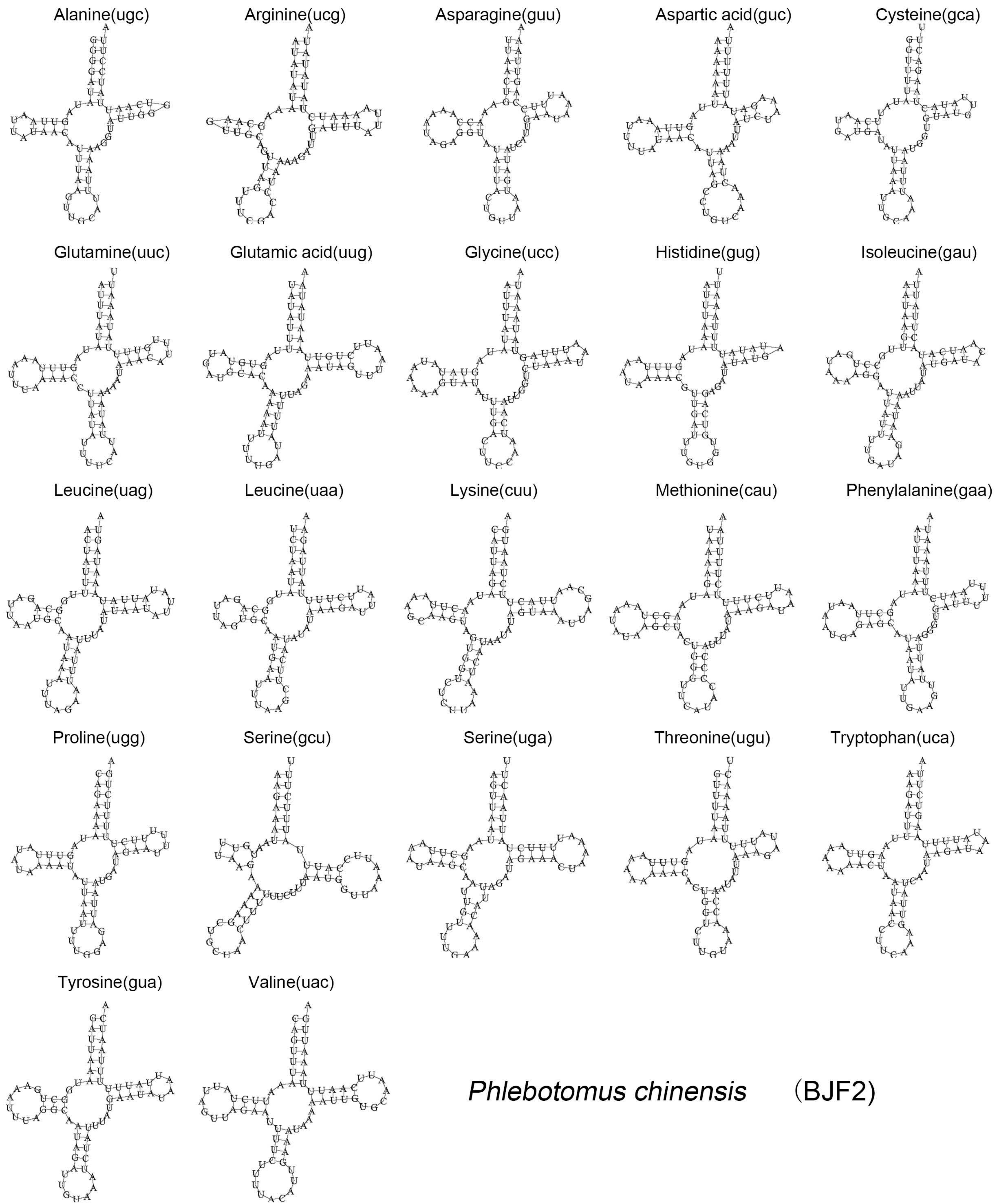

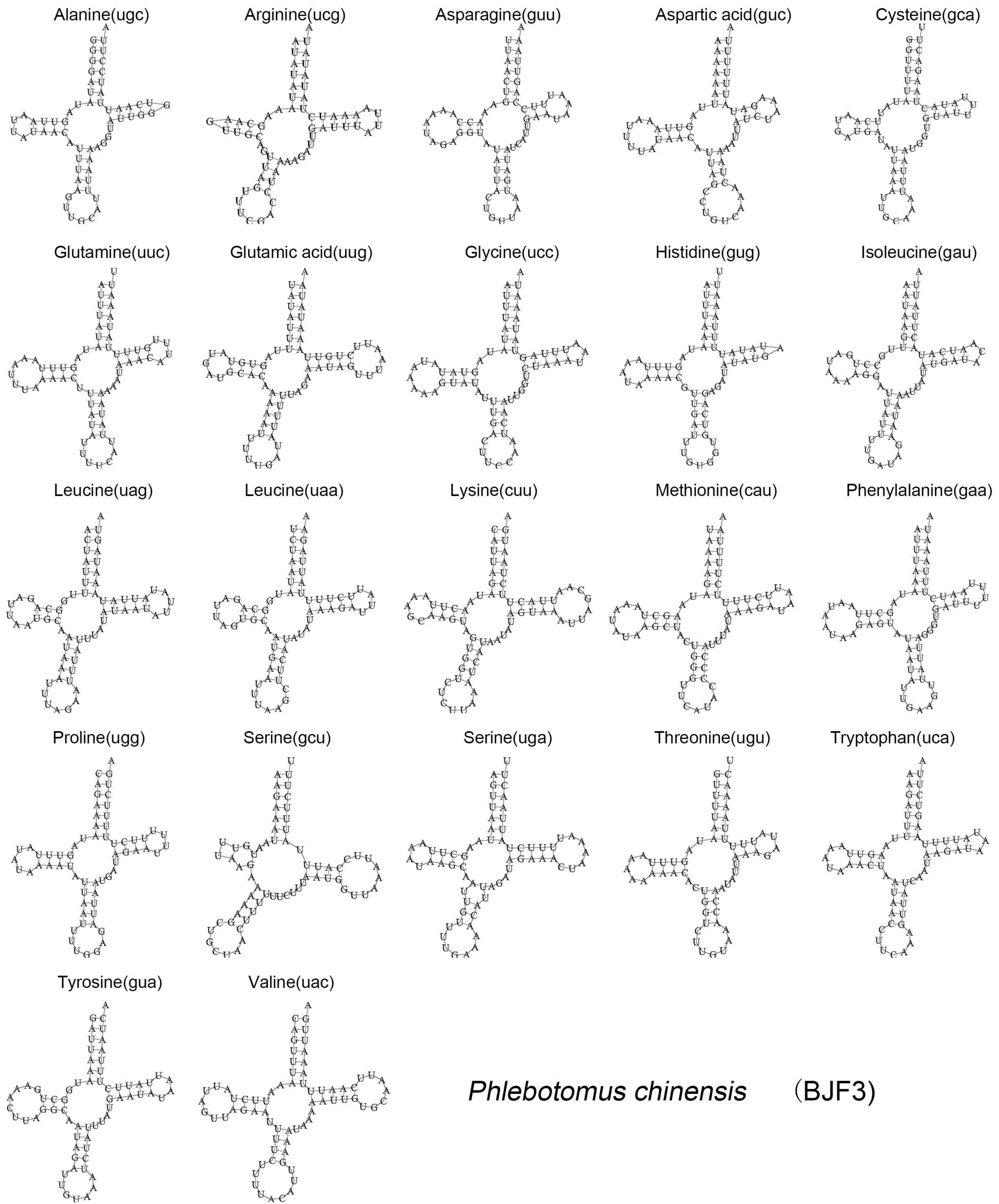

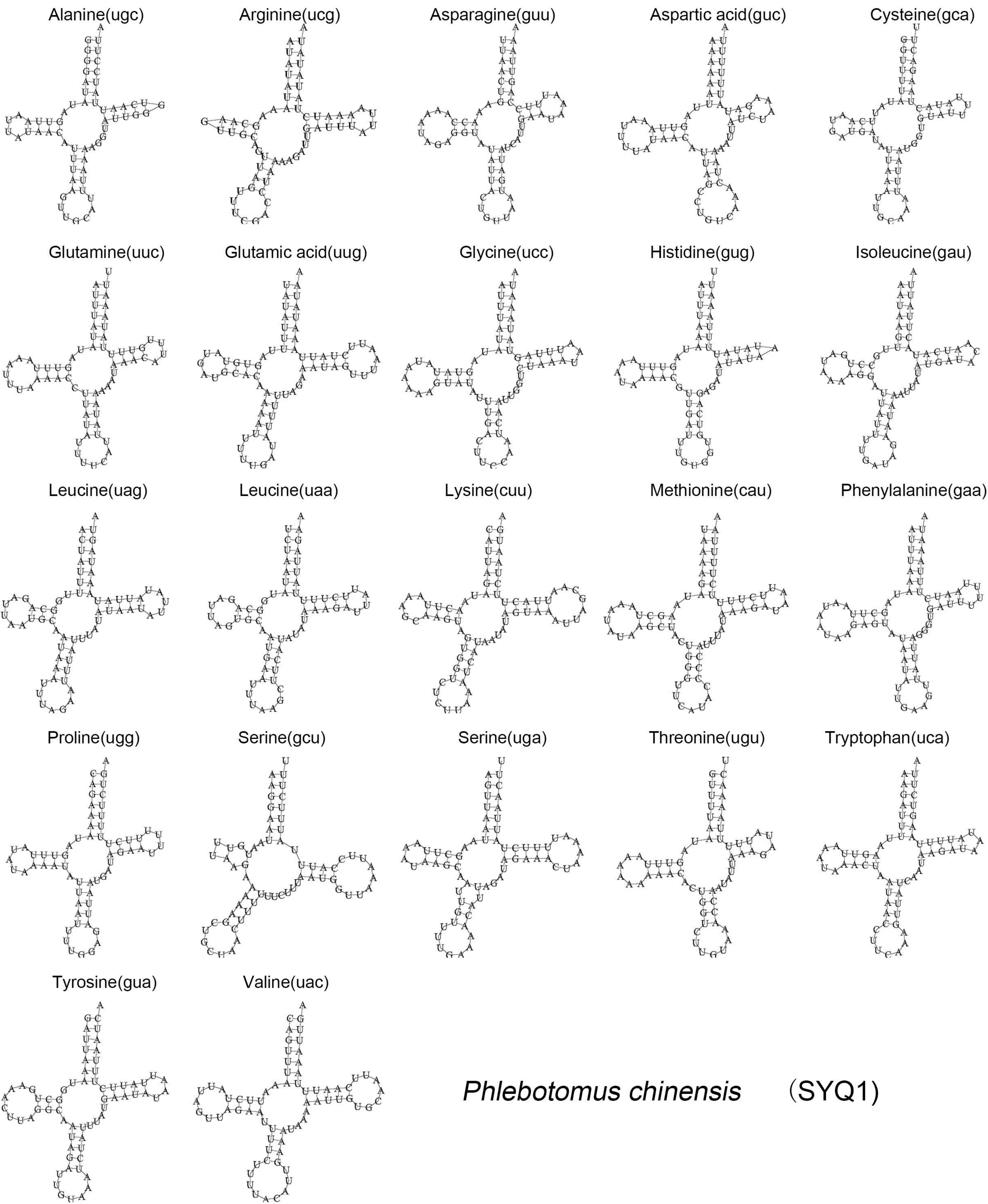

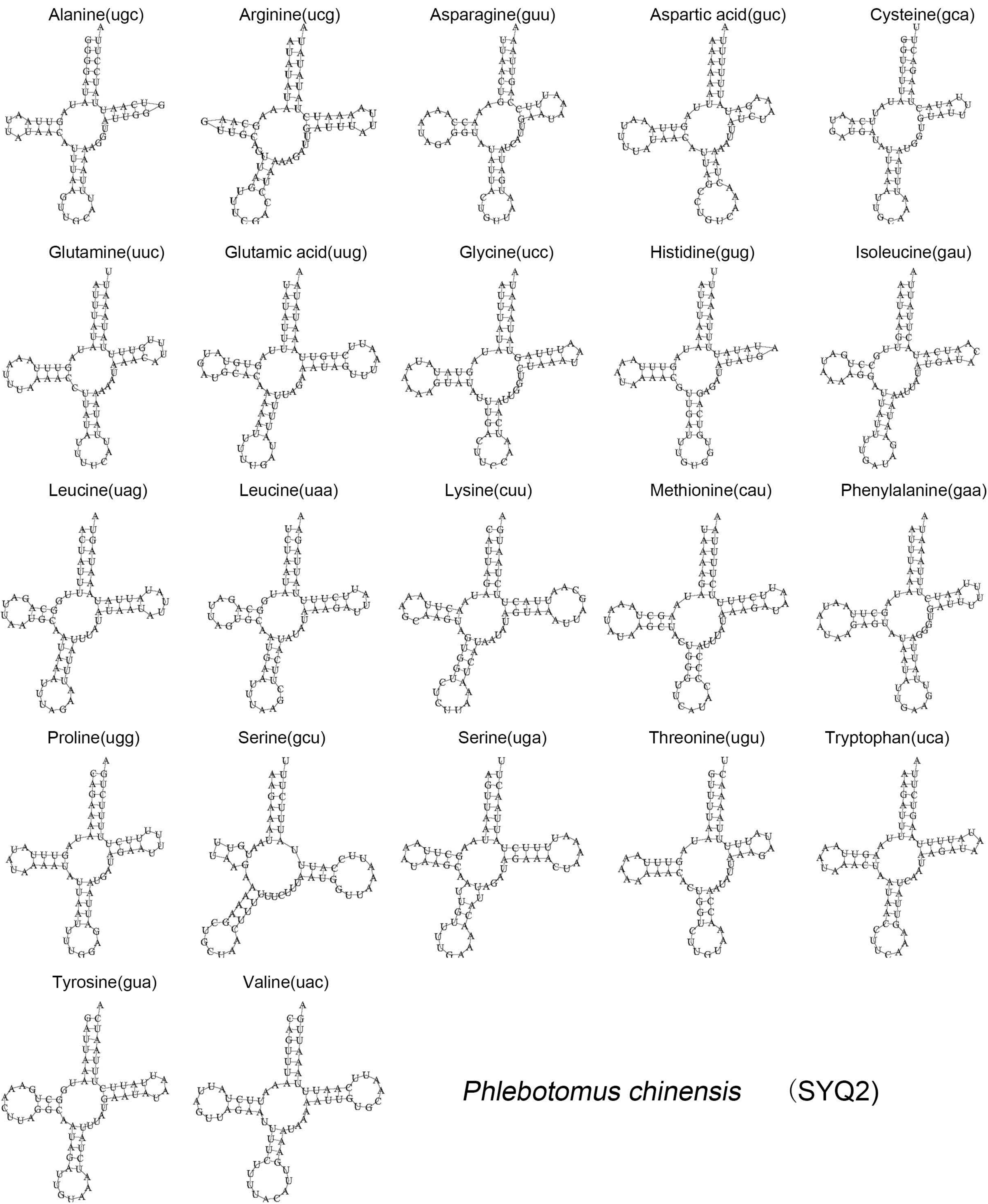

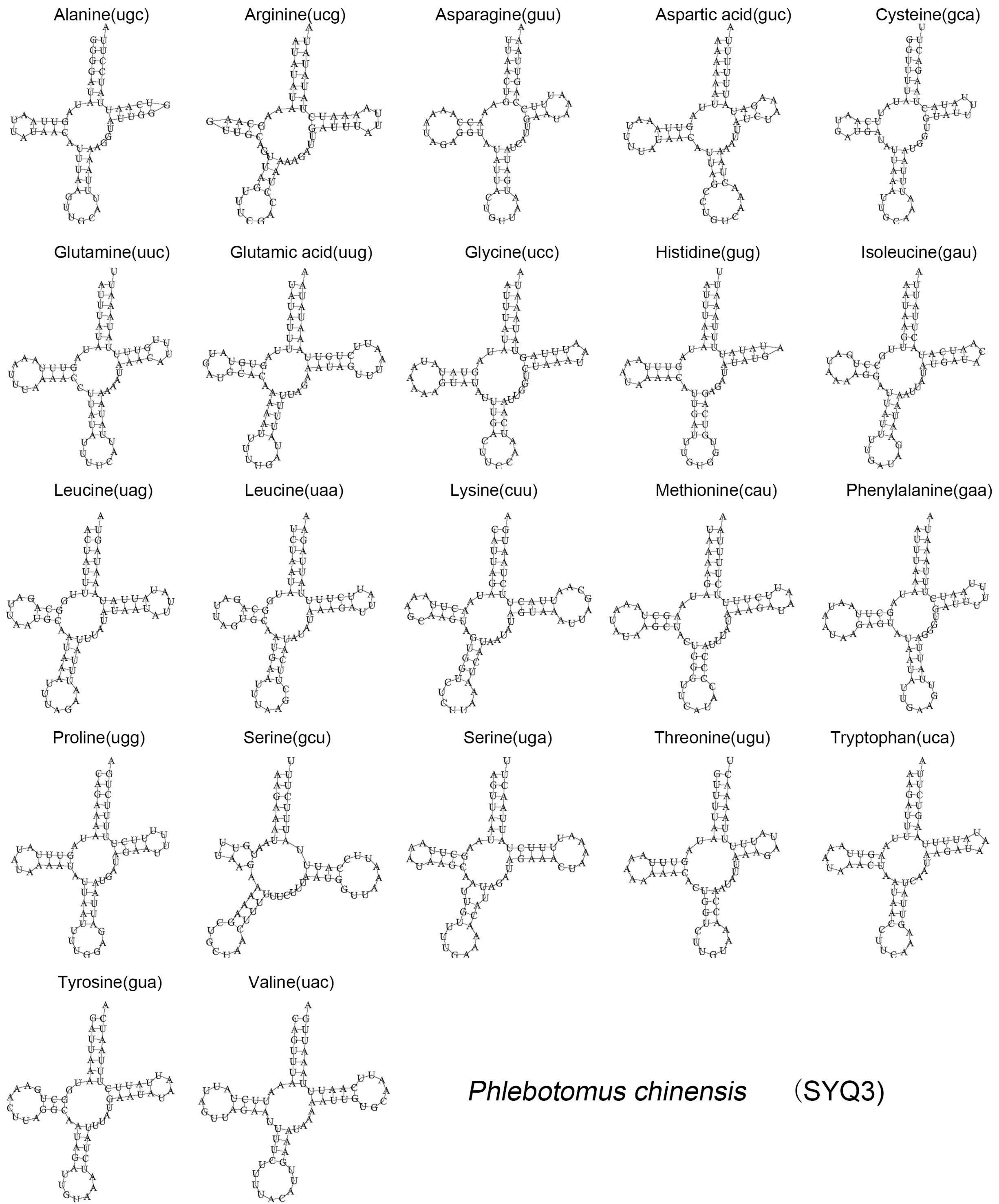

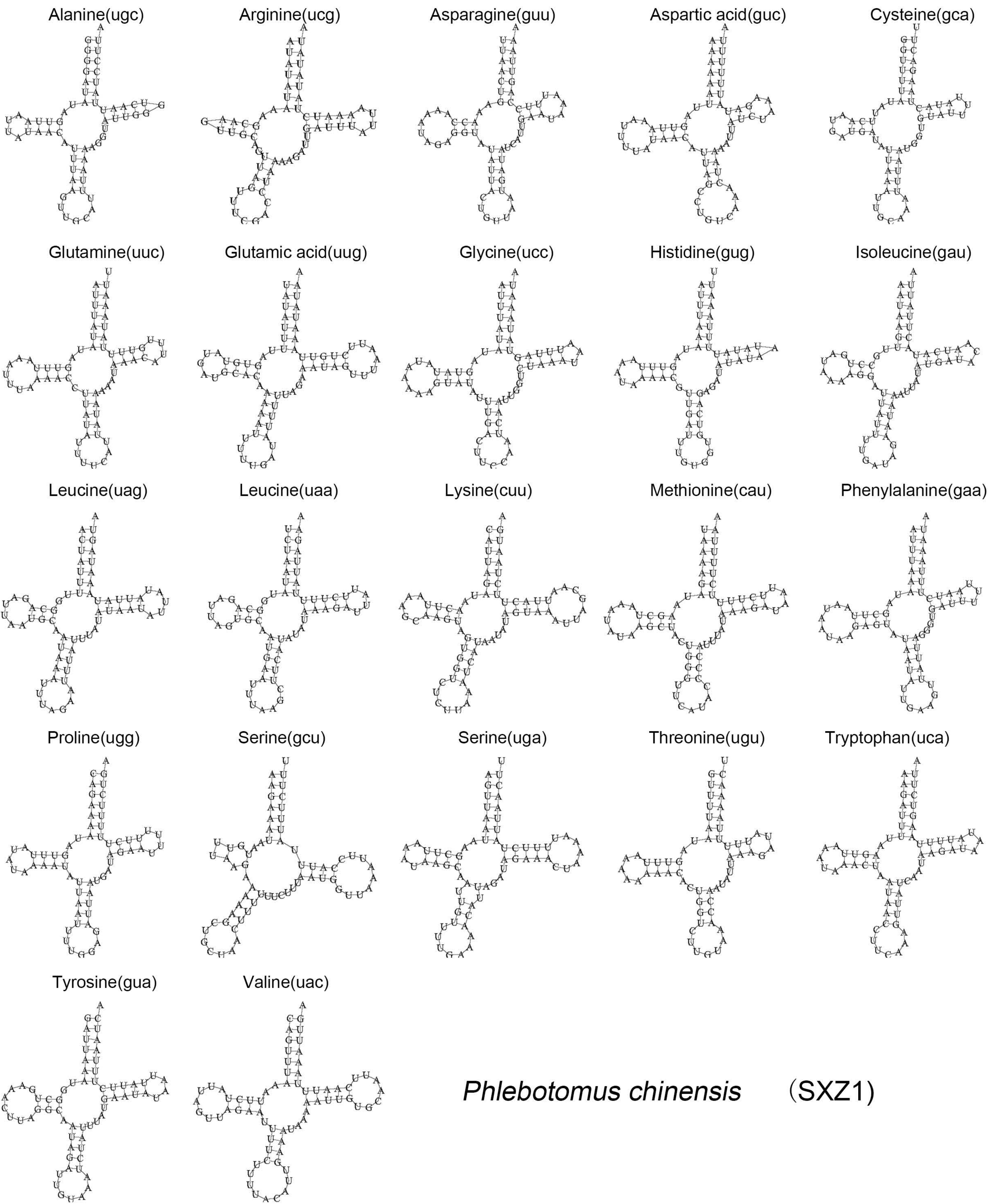

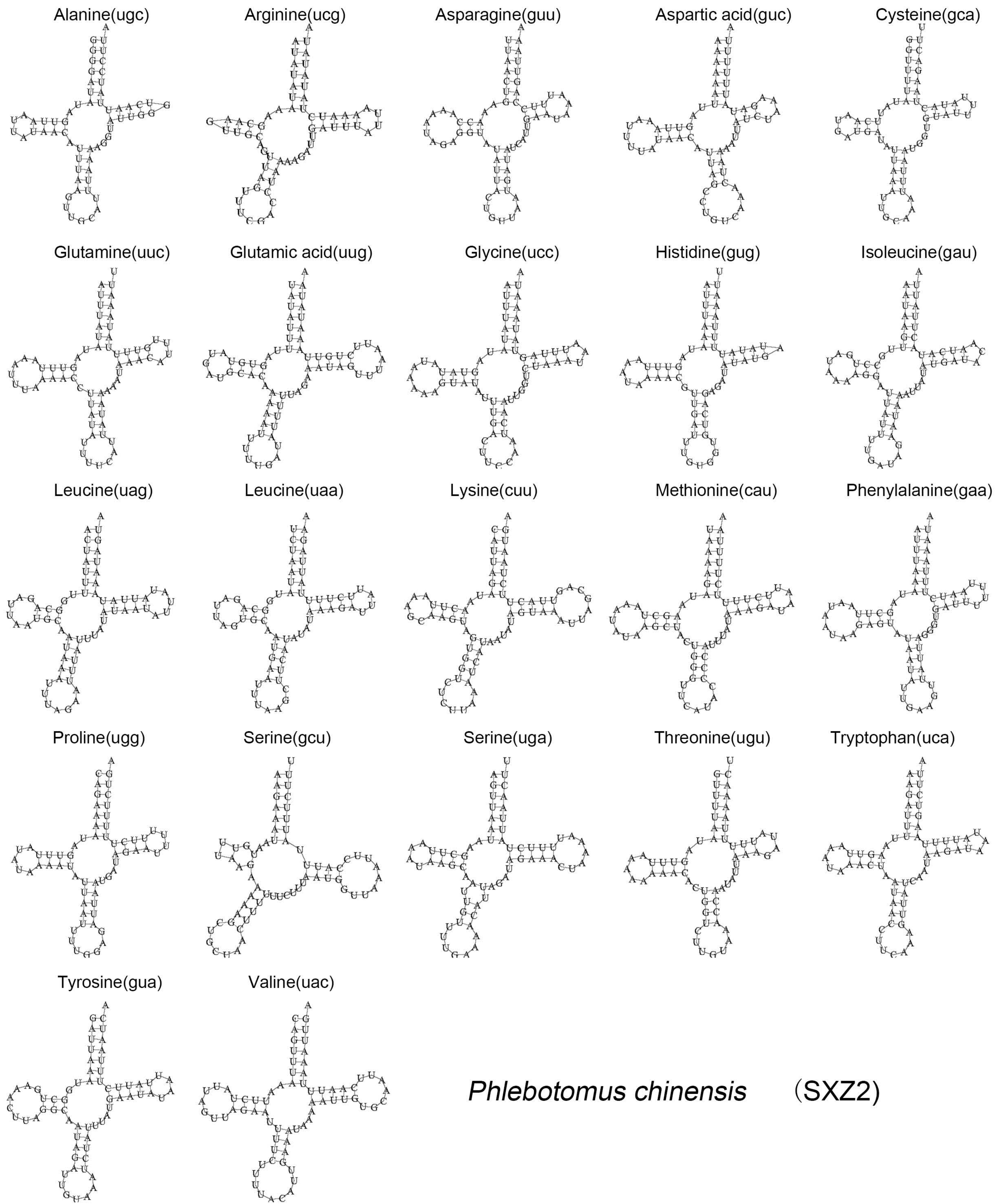

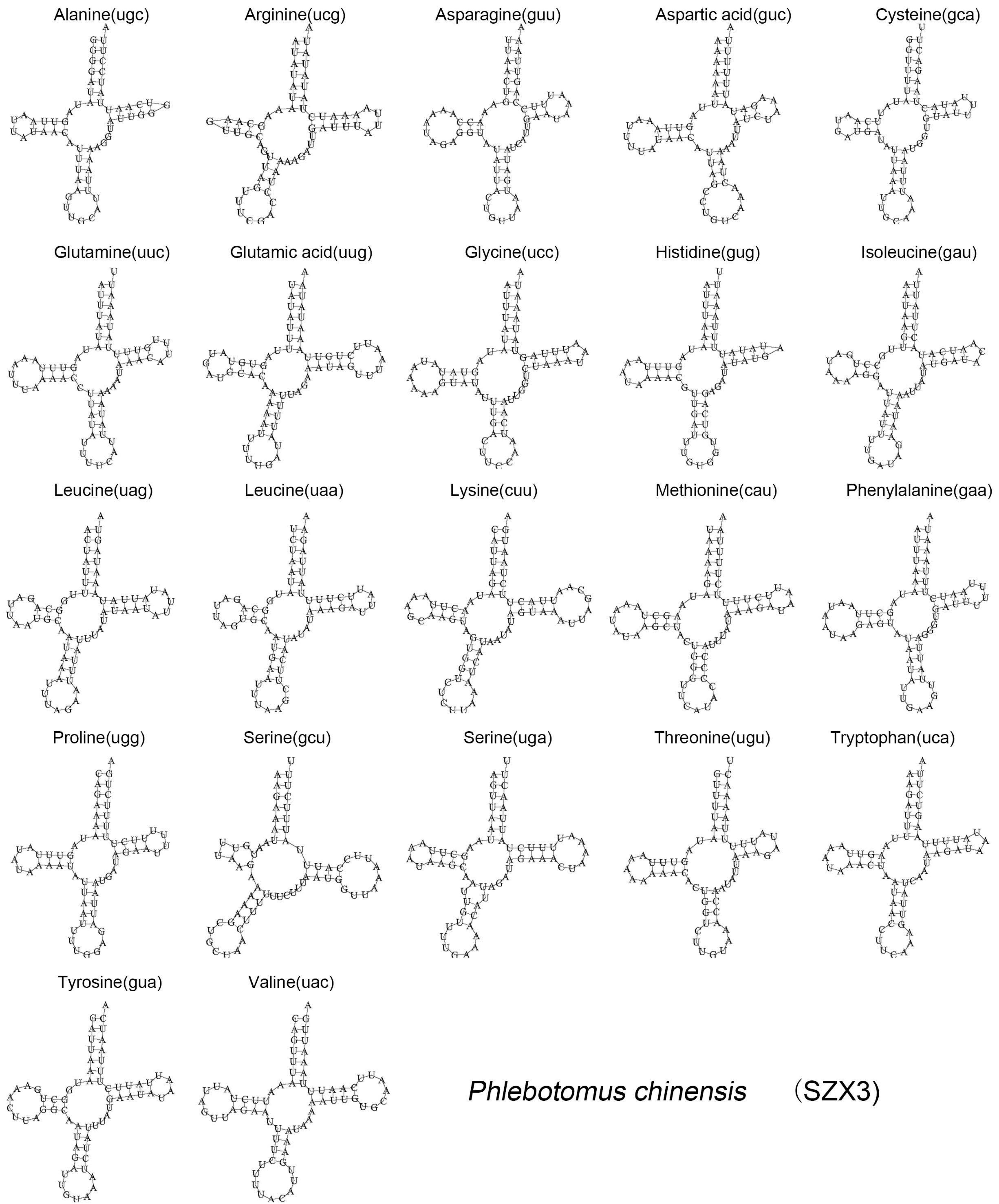

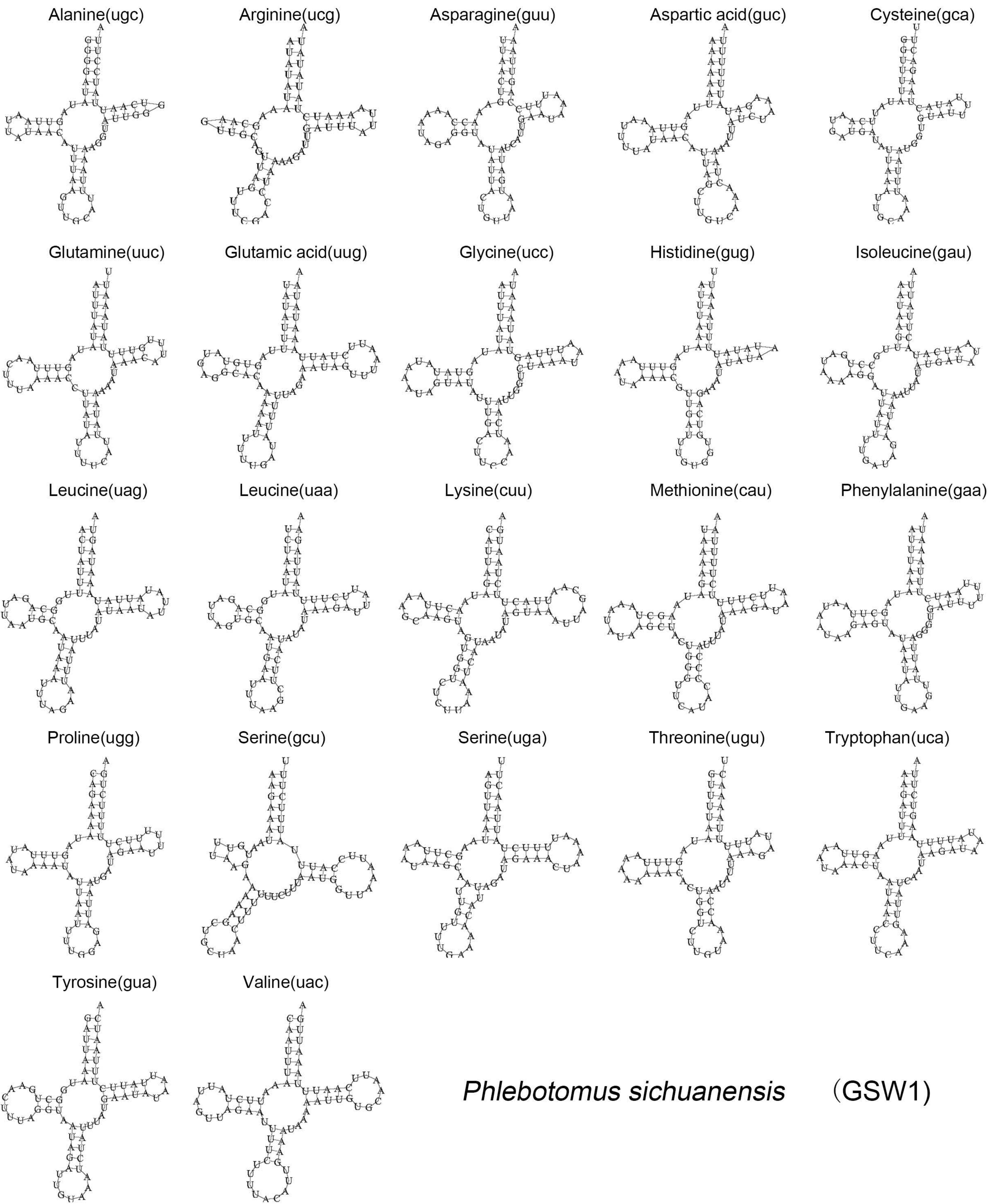

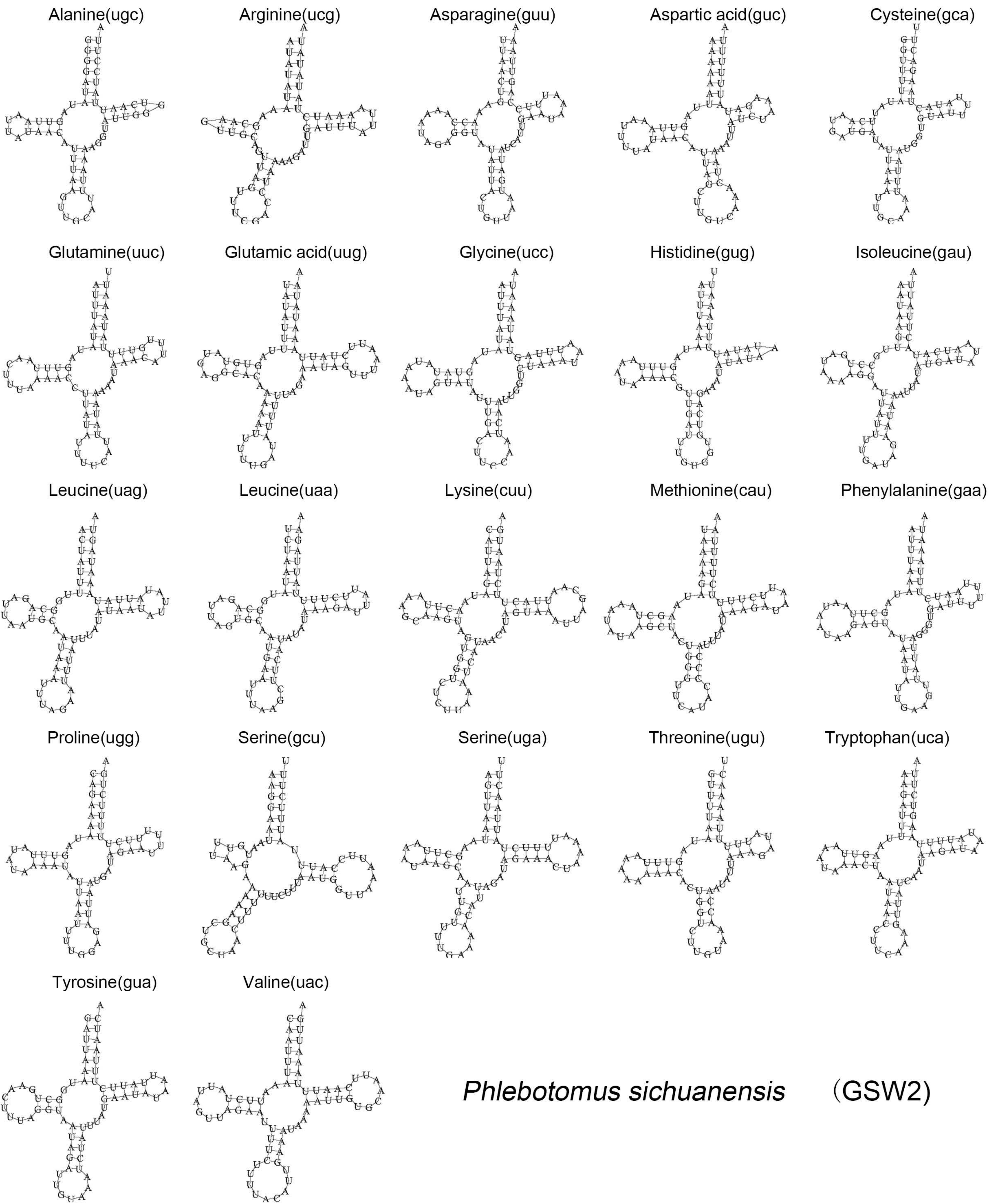

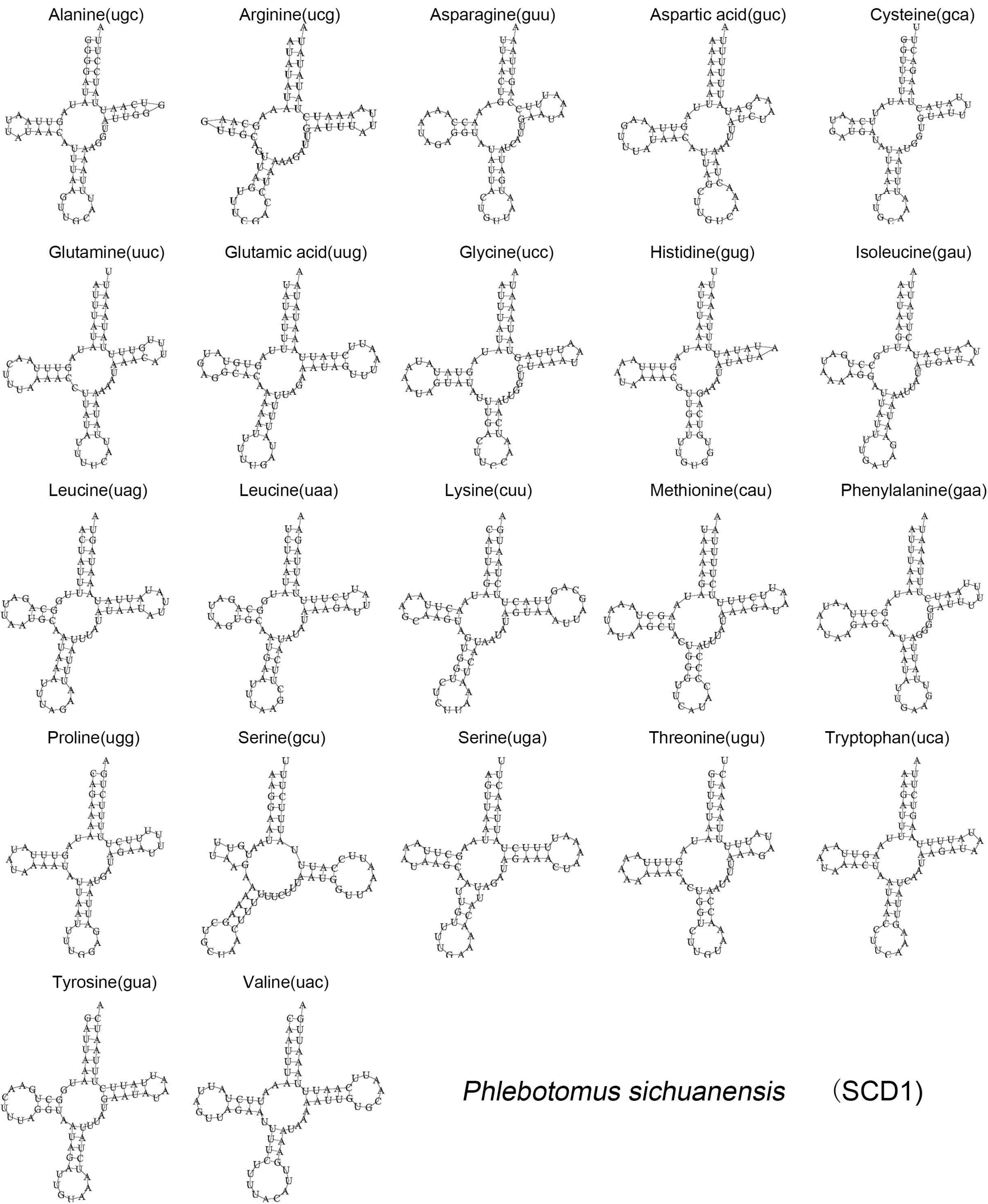

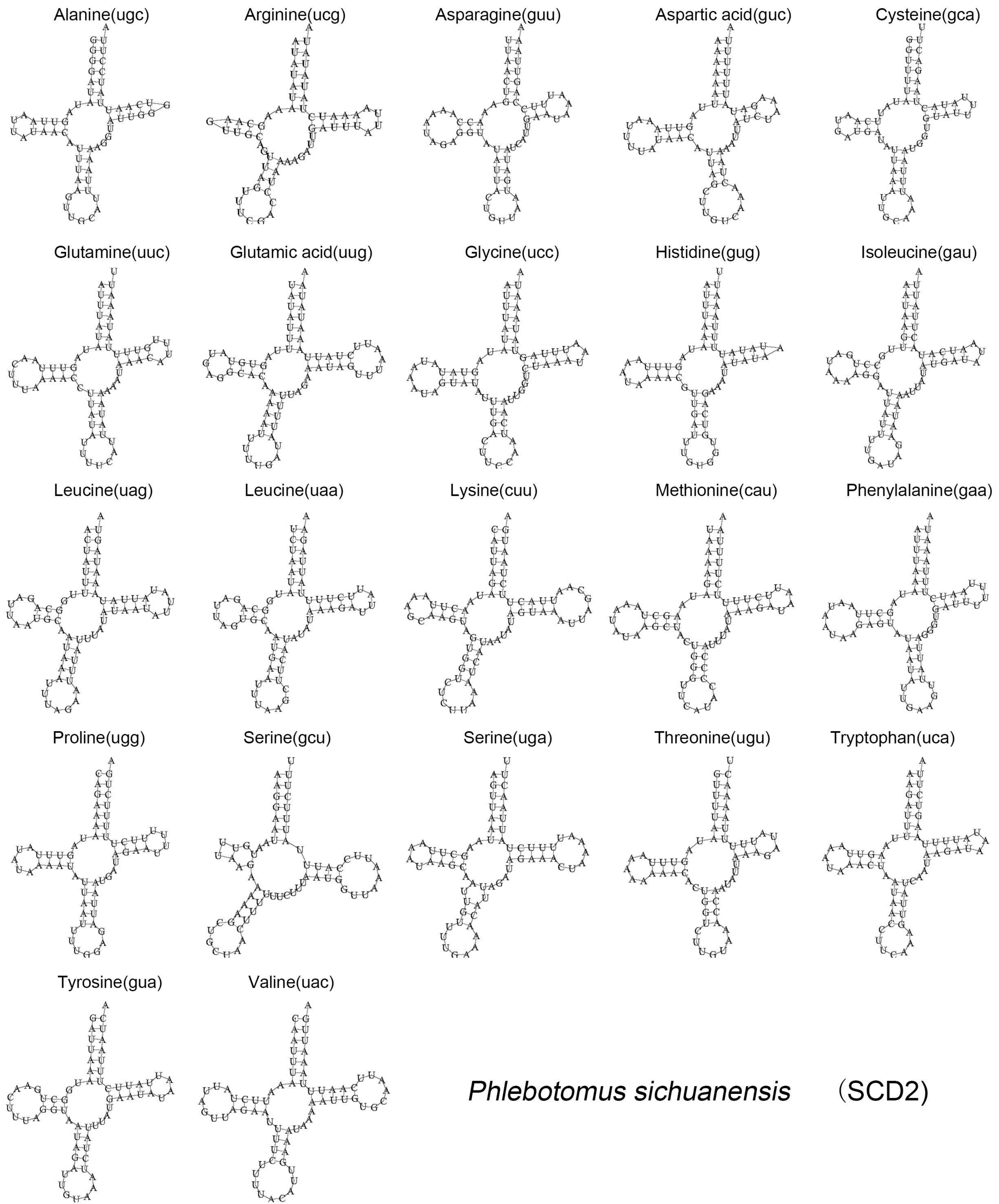

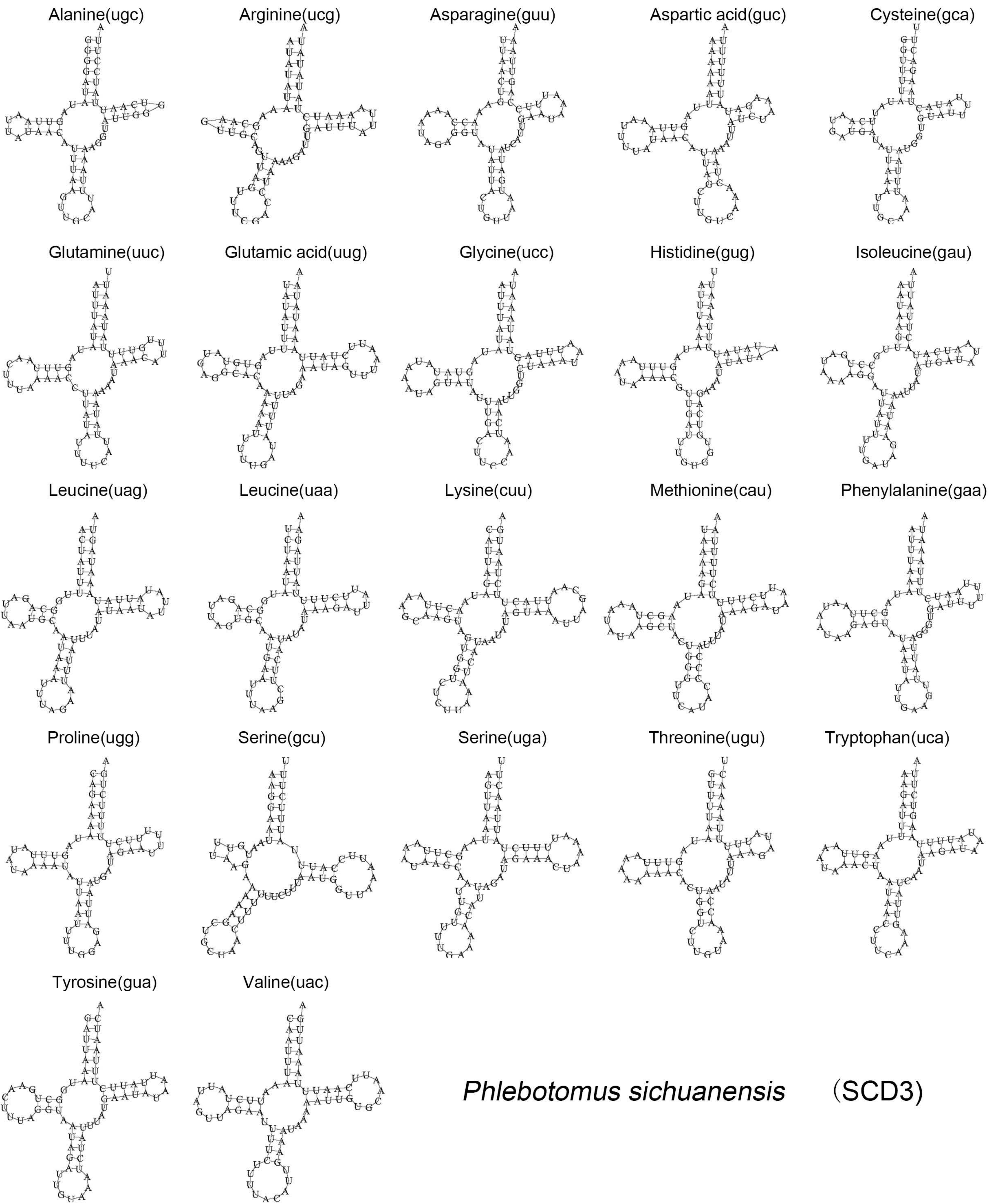

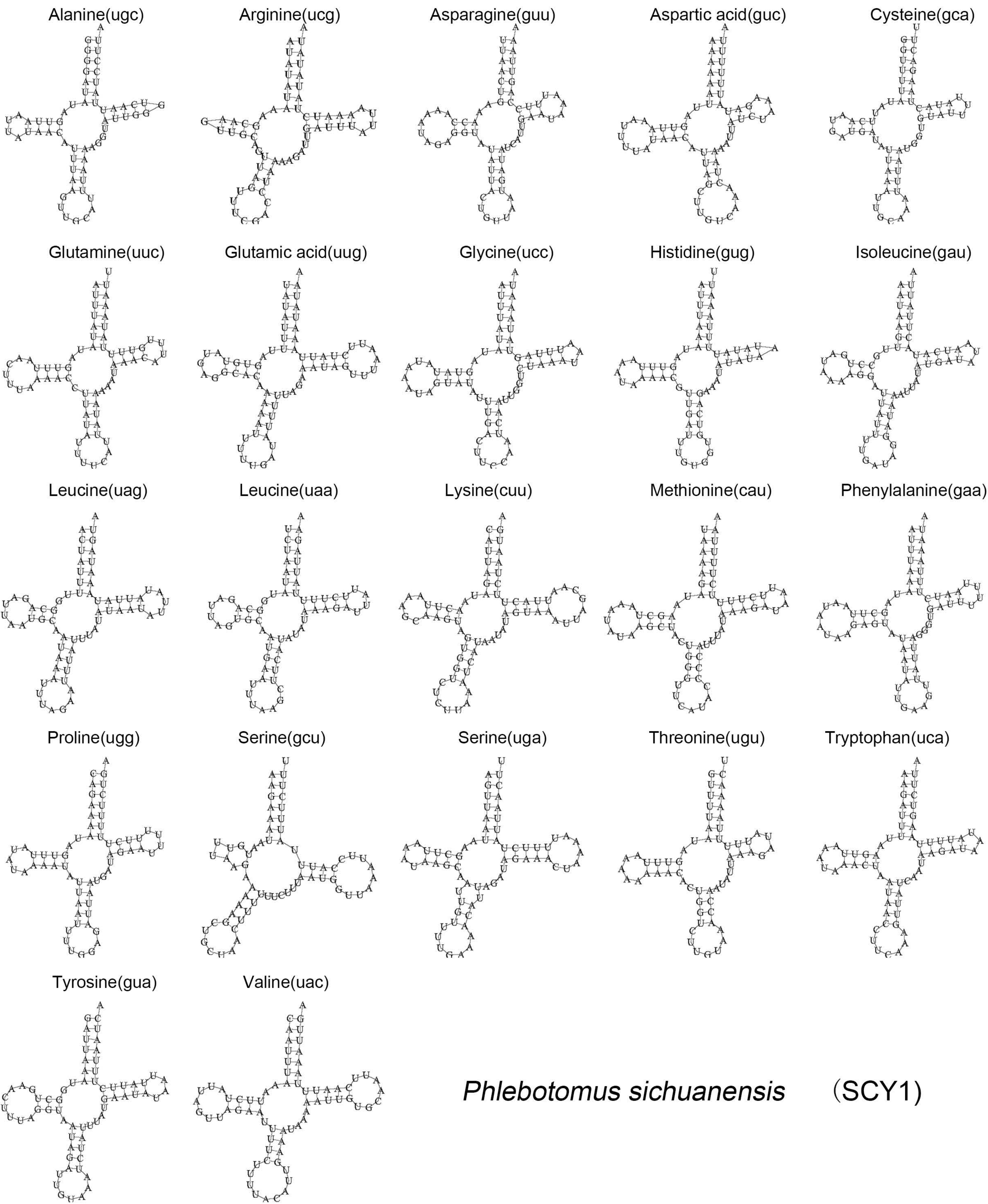

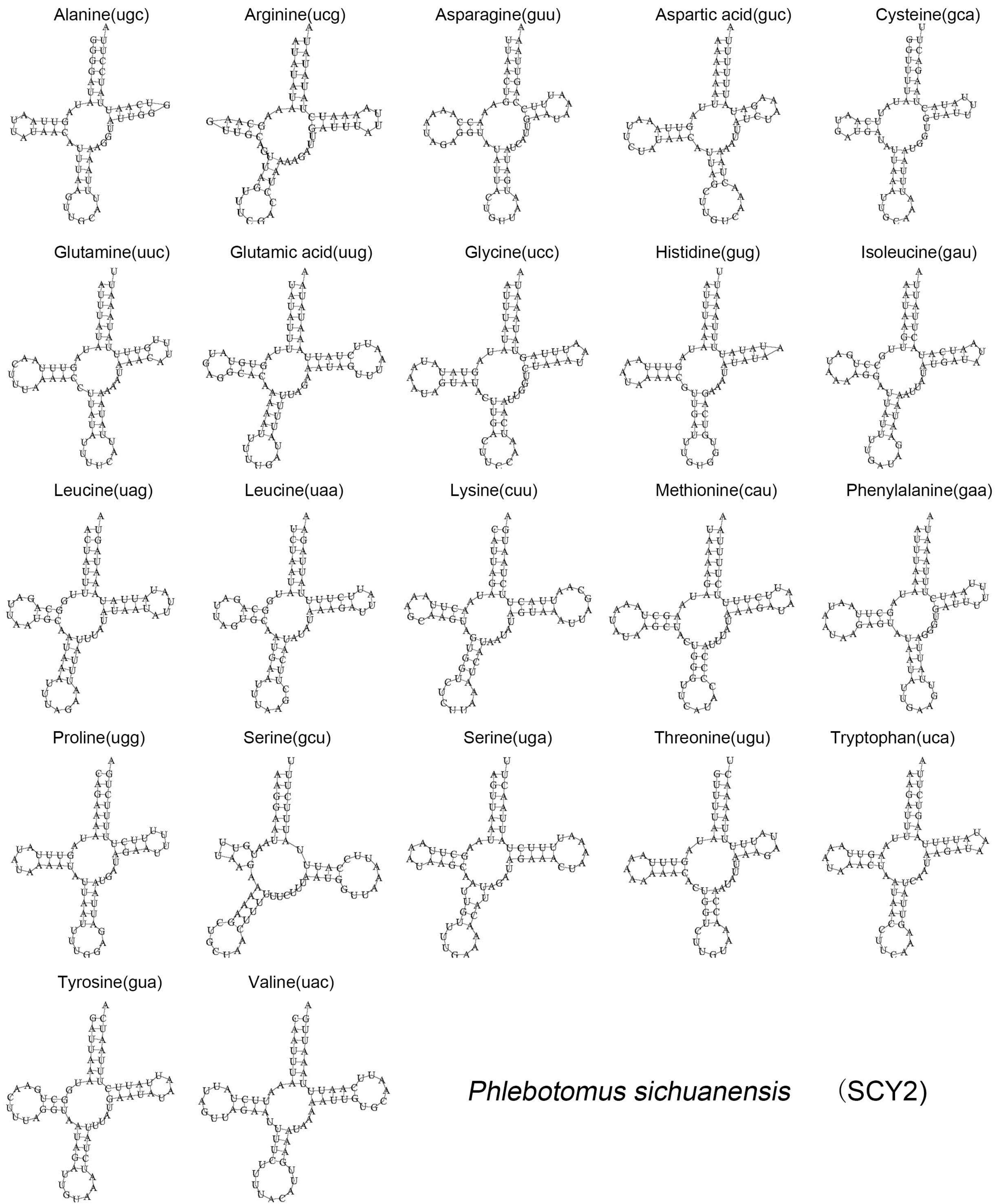

Supplement: Supplementary file 1 [file life-14-01610-s001.zip › Supplementary File 4.pdf]
